# Results of a worldwide survey on the currently used histopathological diagnostic criteria for invasive lobular breast cancer

**DOI:** 10.1038/s41379-022-01135-2

**Published:** 2022-08-03

**Authors:** Maxim De Schepper, Anne Vincent-Salomon, Matthias Christgen, Karen Van Baelen, François Richard, Hitoshi Tsuda, Sasagu Kurozumi, Maria Jose Brito, Gabor Cserni, Stuart Schnitt, Denis Larsimont, Janina Kulka, Pedro Luis Fernandez, Paula Rodríguez-Martínez, Ana Aula Olivar, Cristina Melendez, Mieke Van Bockstal, Aniko Kovacs, Zsuzsanna Varga, Jelle Wesseling, Rohit Bhargava, Pia Boström, Camille Franchet, Blessing Zambuko, Gustavo Matute, Sophie Mueller, Anca Berghian, Emad Rakha, Paul J. van Diest, Steffi Oesterreich, Patrick W. B. Derksen, Giuseppe Floris, Christine Desmedt

**Affiliations:** 1 Laboratory for Translational Breast Cancer Research, Department of Oncology, KU Leuven, Leuven, Belgium; 2 Diagnostic and Theranostic Medicine Division, Institut Curie, PSL Research University, Paris, France; 3 Institute of Pathology, Hannover Medical School, Hannover, Germany; 4 Department of Basic Pathology, National Defense Medical College Hospital, Tokorozawa, Saitama Japan; 5 Department of Breast Surgery, International University of Health and Welfare, Narita, Chiba Japan; 6 Breast Unit, Champalimaud Clinical Center, Champalimaud Foundation, Lisbon, Portugal; 7 Department of Pathology, Bács-Kiskun County Teaching Hospital, Kecskemét, Hungary & Department of Pathology, Albert Szent-Györgyi Medical Center, University of Szeged, Szeged, Hungary; 8 Brigham and Women’s Hospital, Dana-Farber Cancer Institute and Harvard Medical School, Boston, MA USA; 9 Department of Pathology, Institut Jules Bordet, Université Libre de Bruxelles (ULB), Brussels, Belgium; 10 Department of Pathology, Forensic and Insurance Medicine, Semmelweis University, Budapest, Pest Hungary; 11 Hospital German Trias i Pujol, Universidad Autonoma de Barcelona, Barcelona, Spain; 12 University Hospital Doctor Josep Trueta, Girona, Spain; 13 Department of Pathology, Cliniques universitaires Saint-Luc Bruxelles, Woluwé-Saint-Lambert, Brussels, Belgium; 14 Department of Clinical Pathology, Sahlgrenska University Hospital, Gothenburg, Sweden; 15 Department of Pathology and Molecular Pathology, University Hospital Zurich, Zurich, Switzerland; 16 Divisions of Molecular Pathology and Diagnostic Oncology, Netherlands Cancer Institute, Amsterdam, Netherlands; 17 Department of Pathology, UPMC Magee-Womens Hospital, Pittsburgh, PA USA; 18 Department of Pathology, Turku University Hospital and University of Turku, Turku, Finland; 19 Institut Claudius Regaud, Institut Universitaire du Cancer Toulouse - Oncopole, Toulouse, France; 20 Department of Pathology, Sir Ketumile Masire Teaching Hospital, University of Botswana, Gaborone, Botswana; 21 Clínica Universitaria Bolivariana, Universidad Pontificia Bolivariana, Medellín, Colombia; 22 Department of Biopathology, Centre Henri Becquerel, Rouen, France; 23 Department of Histopathology, Nottingham University Hospital NHS Trust, City Hospital Campus Hucknall Road, Nottingham, UK; 24 Department of Pathology, University Medical Center Utrecht, Utrecht, The Netherlands; 25 Women’s Cancer Research Center, UPMC Hillman Cancer Center, Magee-Womens Research Institute, Pittsburgh, PA USA; 26 Department of Pathology, University Hospitals Leuven, UZ Leuven, Leuven, Belgium

**Keywords:** Pathology, Breast cancer

## Abstract

Invasive lobular carcinoma (ILC) represents the second most common subtype of breast cancer (BC), accounting for up to 15% of all invasive BC. Loss of cell adhesion due to functional inactivation of E-cadherin is the hallmark of ILC. Although the current world health organization (WHO) classification for diagnosing ILC requires the recognition of the dispersed or linear non-cohesive growth pattern, it is not mandatory to demonstrate E-cadherin loss by immunohistochemistry (IHC). Recent results of central pathology review of two large randomized clinical trials have demonstrated relative overdiagnosis of ILC, as only ~60% of the locally diagnosed ILCs were confirmed by central pathology. To understand the possible underlying reasons of this discrepancy, we undertook a worldwide survey on the current practice of diagnosing BC as ILC. A survey was drafted by a panel of pathologists and researchers from the European lobular breast cancer consortium (ELBCC) using the online tool SurveyMonkey®. Various parameters such as indications for IHC staining, IHC clones, and IHC staining procedures were questioned. Finally, systematic reporting of non-classical ILC variants were also interrogated. This survey was sent out to pathologists worldwide and circulated from December 14, 2020 until July, 1 2021. The results demonstrate that approximately half of the institutions use E-cadherin expression loss by IHC as an ancillary test to diagnose ILC and that there is a great variability in immunostaining protocols. This might cause different staining results and discordant interpretations. As ILC-specific therapeutic and diagnostic avenues are currently explored in the context of clinical trials, it is of importance to improve standardization of histopathologic diagnosis of ILC diagnosis.

## Introduction

Breast cancer is one of the most frequently diagnosed cancers worldwide, as one in eight women will get the diagnosis of breast cancer during her lifetime in Western countries^1^. Different histological types exist, accompanied by their specific clinical characteristics. Invasive breast carcinoma of no special type (IBC-NST), formerly known as invasive ductal carcinoma, remains the most frequent subtype (~75%), followed by invasive lobular breast cancer (ILC, ~10–15%)^2^.

Most characteristic of ILC is the infiltrative growth of dyscohesive cells in a single file or, the concentric growth of tumor cells around pre-existing breast structures also referred to as “targetoid pattern” (WHO classification of Tumours 5^th^ edition, breast tumors 2019)^3^. Several other growth patterns and nuclear grades of ILC are also recognized, all sharing cell dyscohesion. The underlying molecular feature responsible for this specific appearance are the loss of function mutations and a subsequent loss of heterozygosity (LOH) of *CDH1*, the gene coding for E-cadherin, and occasionally epigenetic silencing^4,5^. E-cadherin is expressed at the cell membrane where it safeguards epithelial cell-cell adhesion^6^. Loss of E-cadherin protein function thus results in loss of cell-cell adhesion with loss of β-catenin membrane expression and cytoplasmic translocation of p120-catenin leading to the typical morphological appearance of ILC, rendering it a true morpho-molecular entity in breast cancer^7–9^.

From a clinical point of view, ILC has dual features, described as ‘initially indolent but slowly progressive’ by Rakha et al.^10^. Indeed, ILC can relapse even > 10 years after diagnosis of the primary tumor^11^, largely arising in the post-menopausal setting and presenting often as multifocal or bilateral tumors^2^. In addition to frequent bone and liver metastasis, ILC is associated with metastatic dissemination in unusual sites such as peritoneum, gastrointestinal- and urinary tract, leptomeninges, skin, orbit and ovaries^12–15^. Regarding management, ILC is less responsive than IBC-NST tumors to chemotherapy, with low pathological complete response (pCR) rates in the neoadjuvant setting^16^. This may be related to the low proliferation rate in ILC compared to other, grade matched, types of breast cancers. Classic lobular carcinoma in situ (LCIS), a non-obligate precursor of ILC, is considered as a risk factor for developing BC and important differential diagnosis needs to be made with ductal carcinoma in situ (DCIS). Namely, when finding classical LCIS on diagnostic biopsy or in the resection margin of a surgical specimen, a more conservative approach can be maintained in contrast to finding DCIS^17^. Also, correct identification of non-lobular ILC mimickers like polymorphous carcinoma of the breast is clinically relevant because of the different hormone receptor status and the aggressive clinical behavior^18^.

ILC is more difficult to recognize on screening mammograms, leading to underestimation of tumor extent, requiring sometimes more extensive surgery or additional surgery^2^. The detection of ILC has dramatically improved due to the introduction of more sensitive modalities such as magnetic resonance imaging (MRI), contrast enhanced digital mammography and digital breast tomosynthesis in the clinical workup, resulting in more adept patient management and care^19–22^. Recognizing ILC as a type of breast cancer is thus relevant not only for the clinical management of the patients, but also for treatment and follow-up protocols^23^. This is especially applicable to the pre-operative diagnostic setting, since a diagnosis of ILC should prompt MRI which in a high percentage reveals bigger, multifocal and bilateral cancers^22^.

Increasing evidence is available for ILC specific therapeutic avenues. In pre-clinical models, synthetic lethality between ROS1 inhibition in E-cadherin deficient cells has been demonstrated, characterized by antitumor effects on E-cadherin deficient breast cancer cells in vivo after the administration of ROS1 inhibitors (such as crizotinib and foretinib)^24^. This was the rationale for recently launched ILC-specific trials in the primary and advanced setting (e.g., Rosaline, ClinicalTrials.gov Identifier: NCT04551495, and ROLO, ClinicalTrials.gov Identifier: NCT03620643). In the BIG1-98 Trial, the effect of an adjuvant aromatase inhibitor (letrozole) versus the selective estrogen receptor modulator tamoxifen was investigated in post-menopausal patients with early, hormone receptor positive, HER2-negative breast cancer and compared between ILC and IBC-NST. The authors concluded that the benefit of adjuvant letrozole over tamoxifen was greater in patients with ILC^25^. Additionally, treatment targets and markers of treatment resistance might differ between ILC and IBC-NST^5,26,27^. An increased frequency of *HER2* mutations has for instance been reported in patients with ILC, especially in the metastatic setting^26^and preliminary results of prospective trials targeting these mutations have shown a higher benefit for patients with ILC than patients with IBC-NST^28^.

ILC is primarily a histopathological diagnosis based on standard hematoxylin and eosin staining (H&E). According to the WHO-classification of breast tumors (5^th^ edition)^3^, immunohistochemistry (IHC) is not required for the diagnosis of ILC, as a proportion of these tumors express E-cadherin despite the typical morphology of ILC. Nevertheless, many pathologists rely on E-cadherin IHC in routine practice to classify cases as ILC. Although classical ILC, especially when associated with lobular carcinoma in situ, is a straightforward diagnosis on H&E slide, different subtypes and growth patterns exist, making the differential diagnosis with IBC-NST sometimes challenging^7^. Moreover, recent central pathology reviews of randomized clinical trials (MINDACT and West German Plan B) have demonstrated relative overdiagnosis of ILC in local pathological laboratories, as only ~60% of the locally diagnosed ILCs were confirmed by central pathology review^29,30^. However, the interobserver agreement analysis was not the primary aim of these studies, nor were the diagnostic procedures of the local centers captured.

When IHC is used, antibodies recognizing E-cadherin are preferred because loss of E-cadherin supports the diagnosis of ILC, acting as a surrogate marker for the underlying genomic alteration. However, interpretation of E-cadherin IHC can be problematic due to aberrant expression of E-cadherin in some cases of ILC (2–26%, depending on the series and the used antibody clones)^31–34^. This is challenging in diagnostic practice, as aberrant expression patterns may be misinterpreted as positive and, as a result, a diagnosis of IBC-NST could be rendered^35^, eventually precluding patients from ILC-specific care. Additional IHC, using β-catenin or p120-catenin^36^, can be used as additional adjuncts to help assess E-cadherin functionality^35^. Absent or perinuclear staining of β-catenin and absent or cytoplasmic translocation of p120-catenin, would then support the diagnosis of ILC in cases with aberrant E-cadherin staining. ILC cases however will be missed using these conventional tools, in which rare somatic *CDH1* mutations are present that do not affect protein expression but cause attenuation or a lack of trans homotypic interactions. These are the interactions of the extracellular E-cadherin domains between adjacent cells, which is involved in the adherens junctions. Further, the adherens junction may also be compromised by loss of function of other proteins in the complex. Here, detailed IHC supported by sequencing of *CDH1* and other adherens junction genes sequencing will be required for the correct diagnosis. Finally, several clones exist for the aforementioned antibodies, some E-cadherin antibodies also recognize P-cadherin (P.W. Dersken and M. Christgen, personal communication), and there are no clear recommendations or guidelines for the use in diagnostic practice.

In summary, there is a discordance between different pathology labs in terms of diagnostic preferences and techniques for ILC, leading to a clinically relevant problem: under- or overdiagnosis of ILC. The diagnosis of ILC (and LCIS) is of importance as it is associated with specific care modalities and future specific therapies. In order to understand the variable practices in ILC diagnostics at the histopathological level, we undertook a worldwide survey on the currently favored histopathological diagnostic preferences and criteria for the diagnosis of ILC.

## Materials and methods

### Survey

An online survey was drafted by a panel of pathologists and researchers from the European lobular breast cancer consortium (ELBCC) using SurveyMonkey®. This survey was circulated by email to pathology laboratories all around the world and was open for entries from December 14, 2020 until July, 1 2021. Pathologists were invited through the pathology network of ELBCC/Lobsterpot and their international connections, through coordinators of cancer hospitals/oncology departments and through larger cancer organizations. The main goals were to register the reporting of ILC diagnosis, with its variants, the use of ancillary IHC use of E-cadherin as a diagnostic marker for ILC and the systematic reporting of the ILC subtypes. A total of 42 questions were asked, with emphasis on use of IHC. First, 7 questions were asked regarding the setting and institution the respondent works in. Next, 4 questions were asked about the indication for performing IHC in clinical practice. Finally, we sought to delineate E-cadherin, β-catenin and p-120 catenin IHC use in more detail. For each antibody, participants were asked about their respective antibody clone, concentration, modality of antigen retrieval and tissue of validation. The list of questions from the survey can be found in the Supplementary Table 1.

### Statistical methods

Associations between categorical variables were assessed using Fisher exact tests. P values were 2-sided and considered as statistically significant at the conventional level of < 0.05. Correction for multiple testing was not applied given the descriptive nature of the work. The statistical analysis was performed using R 4.0.2.

## Results

### Demographics and characteristics of the survey participants

A total of 153 entries were recorded. 147 unique entries were recorded from 34 different countries spread over 6 continents. Most entries were recorded for Europe (63%, 92/147), followed by Asia (18%, 27/147). The countries with the largest contribution were Japan (12%, 18/147), Belgium (10%, 15/147), France (9%, 13/147) and The Netherlands (8%, 12/147, Table 1).

**Table 1:** Overview of continents and countries of the participants.

**Europe**	**92**
Belgium	15
France	13
The Netherlands	12
Spain	10
Germany	8
Hungary	8
Italy	6
Albania	3
Poland	3
Portugal	2
England	2
Sweden	2
Denmark	1
Austria	1
Croatia	1
Latvia	1
Malta	1
Finland	1
Switzerland	1
Ireland	1
**Asia**	**27**
Japan	18
Vietnam	3
Mongolia	3
China	2
Lebanon	1
**South America**	**11**
Argentina	5
Colombia	4
Brazil	2
**North America**	**11**
USA	9
Canada	2
**Africa**	**4**
Botswana	2
Uganda	1
DRCongo	1
**Oceania**	**2**
Australia	2
**Grand Total**	**147**

The majority of the pathologists declared working in a university hospital (56%, 82/147) or large tertiary hospital (30%, 44/147). The vast majority of the pathologists (110/147, 75%) worked in a place with an average yearly volume of breast cancers above 300, with 80/147 (54%,) even above 500. Most pathologists declared being specialized in breast pathology (78%, 115/147).

Only a minority reported working in a small community hospital (5%, 7/147) or a private laboratory (10%, 14/147). However, most centers reported an average volume of >300 BC samples per year in respectively 57% (4/7) and 93% (13/14) of the centers.

There was no significant difference between the continents concerning the type of institution (academic, large tertiary, private, *p* = 0.14, Supplementary Table 2). There was however a significant difference in the reported yearly average breast cancer samples (*p* = 0.0005), and the number of pathologists that handle breast cancer specimens per center between the continents (*p* = 0.0005). In general, there is a relatively higher proportion of participants from North America handling more breast cancer specimens (>500 per year), or working in an institution with more breast pathologists, in comparison to Europe and Asia (Supplementary Table 2). These results should however be interpreted with caution since some continents only had few participants.

### Practice of ILC diagnostics

**Fig. 1:**
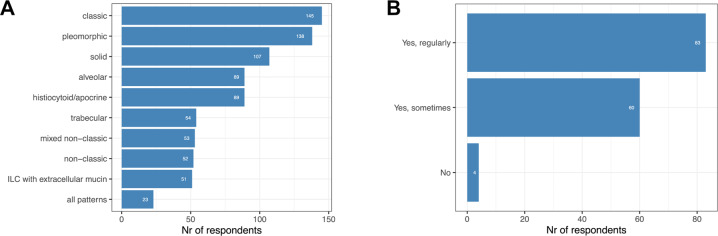
Current practice of ILC diagnostics. **A** Reported patterns of ILC. **B** Use of consensus diagnostics.

Not all lobular variants are systematically reported by all the surveyed pathologists: classic (145/147, 99%), pleomorphic (138/147, 94%), solid (107/147, 73%), histiocytoid (89/147, 61%), alveolar (89/147, 61%), trabecular (54/147, 37%), mixed non-classic (53/147, 36%), non-classic (52/147, 35%) and ILC with extracellular mucin (51/147, 35%), as illustrated in Fig. 1A. Some additional variants that were mentioned are ILC with signet ring cell morphology, ILC with tubular elements and the diffuse type. The latter one is actually a more a radio-pathological term based on the lack of a detectable tumor mass on mammograms (increased density, architectural distortion), and often coincides with the classic histological variant.

Consensus diagnosis, i.e., consulting a colleague/colleagues within the institution for difficult cases, is performed by 97% (143/147) of the participants: 58% reported that consensus diagnosis is regularly done, while 42% reported that consensus diagnosis is only sometimes made. (Fig. 1B).

### Use of IHC for ILC diagnosis

#### Indications

**Fig. 2:**
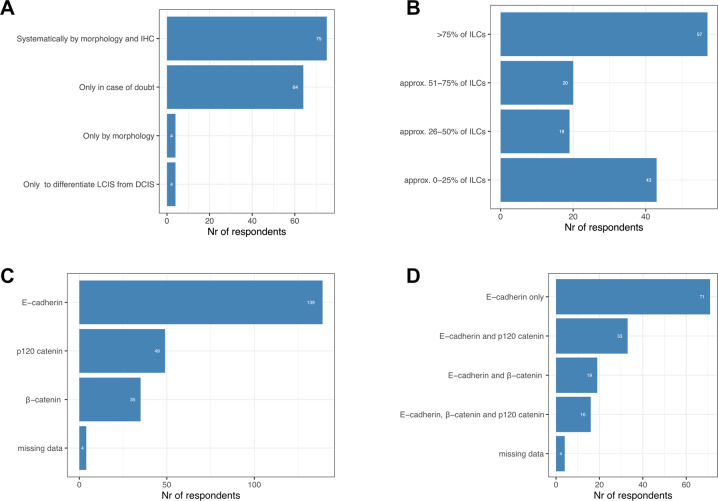
Use of IHC for ILC diagnostics. **A** Indication for using IHC for ILC. **B** Estimated proportion of ILC cases diagnosed by ancillary IHC. **C** Proportion of commonly used antibodies for ILC. **D** Proportion of commonly co-used antibodies for ILC.

The majority of the surveyed pathologists perform IHC for the diagnosis of in situ and invasive lobular neoplasia (97%, 143/147). About half of them systematically perform IHC for ILC diagnosis (52%, 75/143), while others only perform staining in case of doubt (45%, 64/143) or for differentiating DCIS from LCIS (3%, 4/143). Only 3% (4/147) of the pathologists declared to never use IHC for the diagnosis of ILC (Fig. 2A).

When looking at the proportion of cases diagnosed with ancillary IHC, 41% (57/139) reported that > 75% of ILC cases are diagnosed with ancillary IHC while 31% (43/139) reported that this is the case in only 0–25% of ILC cases (Fig. 2B).

Concerning the use of IHC, 139/143 (97%) participants use antibodies recognizing E-cadherin, 35/143 (24%) β-catenin and 49/143 (35%) use p120-catenin (Fig. 2C). The majority (50%, 71/143) uses only E-cadherin, 13% (19/143) use E-cadherin in combination with β-catenin, and 23% (33/143) use E-cadherin with p120-catenin, while 11% (16/143) use all 3 antibodies (Fig. 2D).

Only a minority of the surveyed pathologists uses upfront E-cadherin staining (12%, 16/138), i.e., perform IHC before examination of the H&E slide. We did not observe any significant association between the use or indication of E-cadherin IHC and the type of institution, the volume of breast cancers examined on a yearly basis and the number of pathologists handling breast cancer specimens (Tables 2 and 3).

**Table 2:** Upfront E-cadherin per type of center.

upfront e-cadherin		no	yes	*p*-value
Center of activities	Large tertiary hospital	36 (29.5)	5 (31.2)	0.4533
	Private laboratory	11 (9.0)	3 (18.8)	
	Small community hospital	6 (4.9)	1 (6.2)	
	University Hospital	69 (56.6)	7 (43.8)	
Average volume breast cancer samples/year	0–150 samples	5 (4.1)	1 (6.2)	0.7676
	151–300 samples	23 (18.9)	4 (25.0)	
	301–500 samples	26 (21.3)	3 (18.8)	
	>500 samples	68 (55.7)	8 (50.0)	
Number of pathologist handling breast cancer specimen	1	9 (7.4)	0 (0.0)	0.5802
	1–4	61 (50.0)	10 (62.5)	
	5 or more	52 (42.6)	6 (37.5)

**Table 3:** IHC use per type of center.

		IHC only in case of doubt	IHC only to differentiate LCIS from DCIS	Only by morphology	Systematically by morphology and IHC	*p*-value
Center of activities	Large tertiary hospital	17 (26.6)	1 (25.0)	1 (25.0)	25 (33.3)	0.5802
	Private laboratory	6 (9.4)	1 (25.0)	0 (0.0)	7 (9.3)	
	Small community hospital	3 (4.7)	1 (25.0)	0 (0.0)	3 (4.0)	
	University Hospital	38 (59.4)	1 (25.0)	3 (75.0)	40 (53.3)	
Average volume breast cancer samples/year	0–150 samples	6 (9.4)	0 (0.0)	1 (25.0)	1 (1.3)	0.1259
	151–300 samples	9 (14.1)	2 (50.0)	1 (25.0)	17 (22.7)	
	301–500 samples	16 (25.0)	0 (0.0)	0 (0.0)	14 (18.7)	
	>500 samples	33 (51.6)	2 (50.0)	2 (50.0)	43 (57.3)	
Number of pathologist handling breast cancer specimen	1	5 (7.8)	0 (0.0)	1 (25.0)	4 (5.3)	0.2804
	1 to 4	30 (46.9)	3 (75.0)	0 (0.0)	41 (54.7)	
	5 or more	29 (45.3)	1 (25.0)	3 (75.0)	30 (40.0)	

When a lobular growth pattern is observed, but E-cadherin is positive, 51% (70/138) opt for additional ancillary IHC (β-catenin and/or p120-catenin).

#### Antibodies

##### E-cadherin

For E-cadherin, 11 different antibody clones were reported, of which the NCH-38 was the most frequently used (42%, 38/91), followed by Clone 36 (16%, 15/91) and EP700Y (15%, 14/91), as illustrated in Table 4 (For all reported clones in Supplementary table 3). Heterogeneity is reported regarding the used concentration per clone, with up to seven different concentrations for NCH-38, ranging from 1/50 to 1/300 and the Ready to use (RTU) form. Looking at the distribution of the different antibody clones per continent, we see that NCH-38 is frequently reported in all continents, except for Oceania. Clone 36 was not reported in South America and Africa. Clone EP700Y was not reported in Asia, North America and Oceania (Supplementary Table 4). Caution should be used however for these regional differences regarding continents with lower number of respondents. When looking deeper into the European continent, the NCH-38 was represented by 12/20 countries, except for France, which has a representation of 13 respondents. The dominant clone used here was EP700Y.

**Table 4:** Overview of used concentration per most frequently used E-cadherin clone.

Concentration per clone	Number of participants
**NCH-38**	**38 (42%)**
RTU	12 (13%)
1/100	10 (11%)
1/50	4 (4%)
1/200	3 (3%)
1/300	1 (1%)
1/50 to 1/100	1 (1%)
1/170	1 (1%)
1/25	1 (1%)
Missing data	5 (5%)
**Clone 36**	**15 (16%)**
RTU	8 (9%)
0,314 µg/ml	2 (2%)
1/200	1 (1%)
Missing data	4 (4%)
**EP700Y**	**14 (15%)**
1/200	4 (4%)
RTU	5 (5%)
0,314 µg/ml	1 (1%)
1/700	1 (1%)
unknown	1 (1%)
Missing data	1 (1%)
**Clone 36B5**	**7 (8%)**
RTU	3 (3%)
1/100	1 (1%)
1/40	1 (1%)
Missing data	2 (2%)

As for the interpretation of the staining, the most acknowledged pattern is the complete absence of staining (52%, 150/290), followed by a fragmented membranous pattern (38%, 111/290), perinuclear dot-like pattern (33%, 95/290), and complete but very weak membranous pattern (27%, 78/290). Only 17% (49/290) of the participants recognize all of these patterns in ILC diagnostics.

The most frequently used form of antigen retrieval is the heat induced epitope retrieval (HIER) (84%, 76/91). The means however for HIER are varying, with 6 different modalities of which the automated stainer and hot plate are the most frequently used, in respectively 37% (28/76) and 26% (20/76) of institutions. For HIER, the most frequently used buffers are Tris and/or EDTA based, with basic pH (79%, 60/76), most of which with predominantly pH > 8,0 (63%, 38/60). There is however a minority using citrate buffer with acidic pH (4%, 3/76, Supplementary Table 5).

For the evaluation of the validity of E-cadherin staining, 65% (91/139) of pathologists use an external control on the slide, whereas 36% (48/139) only use the internal control of the examined tissue. These external control tissues are highly variable, but almost always contain tissues with an epithelial component. 50% are non-tumorous tissues (46/91) containing normal breast, liver, colon, appendix, tonsil, kidney, pancreas and skin tissue. 20% (18/91) involve tumor tissue, of which 94% (17/18) contain a breast carcinoma (IBC-NST and/or ILC). In 27/91 (30%) of the entries, the exact tissue could not be attributed to one of these categories, due to missing or unspecific answers)

##### β-catenin

When considering β-catenin, 4 different antibody clones were reported for use in clinical practice, with clone 14 and β-catenin-1 being the most frequently used (in respectively 53% (20/38) and 32% (12/38)). Similar to E-cadherin, there were up to 8 different concentrations reported for clone 14, ranging from 1/75 to 1/300, including 1.25 µg/ml and the RTU form (Supplementary Table 6A). HIER is again the preferred means of antigen retrieval (86%, 31/36), with different modalities of which automated stainer and hot plate remain the most frequent modalities (respectively 35% (11/31) and 29% (9/31)). The buffer used again is Tris and or EDTA based with basic pH (77% (24/31), Supplementary Table 5).

##### P120-catenin

Twenty-nine entries were recorded for specifications regarding p120-catenin. 4 different clones were reported, of which clone 98 and EP66 are the most frequently used products (respectively 48%, 14/29, and 24%, 7/29). For the most used Clone 98, again 7 different concentrations were reported ranging from 1/150 up to 1/500 with inclusion of 0.076 µg/ml and the RTU form (Supplementary Table 6B). Antigen retrieval was again performed preferably by HIER (79%, 23/29), by means of automated stainer (48%, 11/23) and hot plate (26%, 6/23), with basic Tris and/or EDTA-based buffer (83%, 19/23, Supplementary Table 5).

##### Other used IHC antibodies

Pathologists were given the option to enter antibodies other than the ones offered in the questionnaire, and seven responded. Three different antibodies were given: Cytokeratin 34βE12 (5/7), Catenin delta 1 (D7S2M) (1/7), and P-cadherin (clone 56) (1/7, Supplementary Table 6C).

#### Sequencing for CDH1

In case of normal or discordant features between histological findings and immunohistochemistry, we requested whether sequencing for the E-cadherin encoding gene *CDH1* is performed for the definitive diagnosis. Only 4/120 pathologists replied that they rarely perform *CDH1* DNA sequencing (in less than 5% of ILC cases).

## Discussion

We report the results of the first worldwide survey concerning the diagnosis of ILC in pathology practice, in order to explore the possible explanations for suboptimal consistency in BC classification as ILC. As ILC-specific therapeutic avenues are currently being explored, some of which are already being evaluated in clinical trials, it is of importance to further improve the standardization of the histopathologic diagnosis of ILC. Moreover, because of its peculiar biology, patients with ILC require adapted therapeutic decision and disease monitoring strategies^2,23^.

One of the biggest challenges is establishing a clear definition or gold standard of ILC. The goal of the definition should be the identification of patients that have a tumor with a distinct biological behavior. In current clinical practice this is proxied by histopathological findings, in essence based on morphology, aided by IHC and sometimes molecular findings. We do not know however how cases with ambiguous features behave biologically (e.g., lobular morphology with preserved E-cadherin-catenin complex, or IBC-NST morphology with canonical *CDH1* mutations). Two participants reported that, despite preserved E-cadherin staining, a case would still be considered ILC if it has the morphological appearance of an ILC. This is an important question that was however not systematically asked to the pathologists in this survey. It is thus also of utmost importance to investigate which features are indicative and/or decisive for recognizing a tumor as ILC.

In the WHO classification of breast cancer (3), several growth patterns or cellular variants are described with an emphasis on classic ILC and pleomorphic ILC. The most commonly mentioned in a descriptive manner are the solid, the alveolar, the apocrine or histiocytoid variant, and the ILC with signet ring cell features. A tubulolobular variant is mentioned, which is most likely not a variant of ILC, not to be confused with an ILC with tubular elements^37^. We know however that there are more patterns described in literature and also recognized in this survey. The importance of this subtyping has been demonstrated in several studies. First, a prognostic significance has been linked to these variants with a favorable prognosis of the pure classic subtype in comparison to non-classical subtypes^11^. Second, it has been demonstrated that these subtypes or variants display a different molecular landscape. The mixed non-classic subtypes are enriched for *ERBB2* and *TP53* mutations, and frequently present with 1p36.22 (ARID1A) deletions. The solid subtype is characterized by more *ARID1A* mutations, 1p36.22 (ARID1A) deletions and by 11p and 6q25.1 (ESR1) gains. Last, the alveolar subtype is characterized by gains in 11q13.3 (CCND1) and 11q14 (PAK1)^27^. Third, it has become clear that some of these subtypes that are not included in the WHO classification, are a source of misdiagnosis of ILC, such as the trabecular subtype or ILC with tubular elements^37,38^. Remarkably, the majority of pathologists take note of the different variants and growth types of ILC, despite most of them are not considered for clinical decision making.

Our results demonstrate that half of the institutions systematically perform ancillary IHC, mainly E-cadherin based, in order to support the morphological diagnosis of ILC, a practice which is however not mandatory based on the current WHO essential diagnostic criteria^3^. Interestingly, the diagnostic accuracy of ILC between diagnosis based on H&E only and H&E supported with E-cadherin IHC has been recently investigated in a large international study. Here, the investigators demonstrated a significantly increased inter-observer agreement when pathologists were provided with additional E-cadherin IHC, of note, when the antibody clone and staining procedure was equal for all reviewers^38^.

There is however a great variability in E-cadherin antibody clones used as well as their staining procedures (concentration, antigen retrieval modalities, validation), which might result in differences in staining results and their interpretation. There seems to be little heterogeneity between the continents, with most antibody clones for E-cadherin being represented in most continents, with subtle differences between continents concerning presence of a single antibody clone or the reported frequency (Supplementary Table 4).

The most straightforward pattern of E-cadherin staining, is the absence of staining. However, different aberrant patterns are described and used for E-cadherin in clinical practice: cytoplasmic; dot-like, perinuclear Golgi-type pattern; fragmented, focal, or beaded membranous expression; a complete, but weak membranous E-cadherin staining^33^. These patterns however could be dependent on the antibody clone and concentration that is used. Adding the extra variable of different antigen retrieval modalities, results in a complex situation allowing high variability of staining results and subsequent interpretation. Also, it has become clear that false positive E-cadherin staining could be observed, as there exist different somatic mutations in *CDH1* (frameshift mutation, extracellular truncating mutation, extracellular missense mutation, truncating mutation)^38^, and as each antibody may have its own specificity for detecting these mutations. In these cases, although partial membranous E-cadherin staining may be observed, the protein is most probably non-functional. The exact staining pattern, sensitivities and specificities have not been systematically investigated, but are probably of importance.

Concerning the validation of E-cadherin, healthy tissues are mostly used for the presence of a membranous staining in epithelial structures, however the diagnostic performance of the antibody (i.e. being able to reliably distinguish a case between IBC-NST and ILC) is only validated by a minority of the participants (11%, 10/91) and not systematically reported for all antibodies (E-cadherin, p120-catenin and β-catenin). When interrogating for instance the Nordic immunohistochemical Quality Control (NordiQC, 9000 Aalborg, Denmark) reports of E-cadherin assessment, Clone EP700Y (the third most frequent clone used in this survey) underperformed because of strong background staining in normal tissues and non-specific staining in plasma cells in the lamina propria of the colon^39^. However, it is mentioned that the staining still allowed pathologists to distinguish ILC reliably from IBC-NST on the given Tissue Microarray. NordiQC reported that one of the causes of insufficient staining for E-cadherin is the use of an acidic buffer, which is reported by a minority of our participants (4%, 3/76)^39^. It is however not reported if the diagnostic performance of differentiating ILC from IBC-NST was still preserved under these conditions.

The added value of systematic concomitant catenin (p120- or β-catenin) staining or catenin staining alone without E-cadherin is not really recommended, as loss of membranous staining of catenins can be observed in IBC-NST^40^ and in non-breast malignancies^41^. The latter may reflect a state of dedifferentiation or epithelial to mesenchymal transition, rather than a pathognomonic mutation in *CDH1*.

Given the large number of participating countries and different continents, we believe that the present survey provides a rather fair representation of ILC diagnostic practices worldwide. Of note, there might be a bias given the relative overrepresentation of European countries and overrepresentation of Japan within the Asian continent, probably resulting in significant differences observed in the characteristics of the participating centers (Supplementary Table 2). North American countries are relatively less well represented in terms of numbers and the respondents mainly belong to large centers with high breast cancer sample volumes (Supplementary Table 2). Nevertheless, indications for IHC staining do not seem to differ in the different types of centers (Tables 2 and 3). Moreover, the gradual increase of missing data along the course of the survey, as exemplified by the smaller number of pathologists answering the questions related to the use of IHC for ILC (i.e. the last questions in the survey; figure2), may render these entries less reliable in terms of data interpretation.

Within the European Union, all commercially available antibodies for clinical use will need to be compliant with the in vitro diagnostic medical devices regulation (IVDR). The IVDR is already going into effect by 26 May 2022, and foresees a conditional transition phase until 26 May 2024, when all medical devices are required to be compliant to the IVDR. Not all manufacturers have obtained a CE-label yet for the reported antibody clones, and it is unsure if all of them will pursue the acquisition of it. Laboratories would be still be able to use a product without CE-IVD label or a CE-IVD product with deviation of the provided protocol. In these cases however, laboratories would need to provide an extensive validation report of this product and/or protocol (laboratory developed test (LDT)). Given the heterogeneity of staining procedures reported in this survey, it is also unsure whether laboratories will pursue the validation of an LDT or comply to the CE-IVD protocol. The antibody market is thus highly likely to change in the near future. It is however very important to keep the issues mentioned in this manuscript in mind for the acquisition of CE-label.

To conclude, this is the first study to our knowledge to monitor the diagnostic practices for ILC across the world. We have demonstrated possible explanations for the reported discordances in ILC diagnosis such as guidelines open for variable interpretations for diagnosis of ILC and heterogeneity in antibody staining procedures, particularly for E-cadherin. Standardization of IHC staining procedures as well as their interpretation is thus warranted. Pathologists active in breast pathology need to be aware of these existing pitfalls, and discordances between morphology (H&E) and IHC are a strong indication for false positive E-cadherin staining or the presence of a less known ILC variant. Initiatives are ongoing within the ELBCC to further investigate the staining patterns of the most frequently reported E-cadherin clones in this survey according to different types of mutations in *CDH1*, as a further step in streamlining diagnostics, clinical research and patient specific care for ILC.

## Supplementary information

Supplementary Tables

## Data Availability

Data are available as Supplementary Tables 1-6: Supplementary Table 1: survey questionnaire. Supplementary Table 2**:** characteristics of participants per continent. Supplementary Table 3: Overview of used concentration per used E-cadherin clone. Supplementary Table 4: E-cadherin antibody clone per continent. Supplementary Table 5: staining procedures per antibody. Supplementary Table 6**:** concentration per reported clone.
