# Supplementary material for: Results of a worldwide survey on the currently used histopathological diagnostic criteria for invasive lobular breast cancer
Source: Mod Pathol. 2022 Aug 3;35(12):1812–20. doi: 10.1038/s41379-022-01135-2 (PMC9708574; doi:10.1038/s41379-022-01135-2)
Supplement: Supplementary file 1 — Supplementary Tables [file 41379_2022_1135_MOESM1_ESM.pdf]

**Supplementary information for**
**'Results of a worldwide survey on the currently used histopathological diagnostic criteria for invasive lobular breast cancer' by De Schepper et al.**

## Table of Contents


**Supplementary Table 1: survey questionnaire** ........ 2
**Supplementary Table 2: characteristics of participants per continent** ........ 4
**Supplementary Table 3: Overview of used concentration per used E-cadherin clone** ........ 5
**Supplementary Table 4: E-cadherin antibody clone per continent** ........ 7
**Supplementary Table 5: staining procedures per antibody** ........ 8
**Supplementary Table 6: concentration per reported clone** ........ 9
Supplementary Table 6a: concentration per clone for β-catenin ........ 9
Supplementary Table 6b: concentration per clone for p120-catenin ........ 9
Supplementary Table 6c: concentration per clone for other reported antibodies ........ 10

Supplementary Table 1: survey questionnaire

| |
|---|
| **In which country do you practice pathology?** |
| **What is you field of interest in pathology?** |
| **In which center do you perform your activities:** |
| **What is the average volume/year of breast cancer samples in your center?** |
| **How many pathologists handle breast cancer specimens in your laboratory** |
| **Do you perform consensus diagnosis for difficult cases in your center?** |
| **Are you aware of national/international guidelines recommending the use of IHC for the diagnosis of ILC?** |
| **How do you diagnose an invasive lobular carcinoma?** |
| **Which subtypes of ILC do you report? (Multiple options possible):** |
| Classic |
| Non-classic |
| Mixed non-classic |
| Alveolar |
| Solid |
| Trabecular |
| Histiocytoid/Apocrine |
| Pleomorphic |
| Tubulo-lobular |
| Invasive carcinoma with mixed ductal and lobular features |
| Mucinous |
| All of the above |
| Other |
| **What is the proportion of ILCs diagnosed with ancillary IHC in your institution?** |
| **If you perform IHC, which antibody/antibodies do you use?** |
| E-cadherin |
| Beta- catenin |
| p120 catenin |
| Other (specify later) |
| Double staining (please specify) |
| **Which patterns of staining's do you use to support the diagnosis of ILC by E-cadherin? (multiple options possible)** |
| Complete absence of membranous E-cadherin staining |
| Dot-like, perinuclear Golgi-type pattern |
| Fragmented, focal, or fragmented, focal, or beaded expression beaded expression of E-cadherin |
| A complete, but weak membranous E-cadherin |
| All of the above |
| Other |
| **Do you use upfront E-cadherin IHC for every breast cancer sample?** |
| **When E-cadherin is positive but the H&E pattern is lobular: do you use p120 catenin or / and beta-catenin?** |
| **Which pattern do you consider for ILC diagnosis? (Multiple options possible):** |
| cytoplasmic or negative beta catenin |
| cytoplasmic or nuclear p120 staining |
| Do you use an external control for staining? |
| **If you use E-cadherin, what are its specifications?** |
| Clone: |
| Firm: |
| Concentration: |

| |
|---|
| Tissue for validation: |
| **How do you perform antigen retrieval for E-cadherin?** |
| **If you perform HIER, how is it performed?** |
| **Which buffer is used?** |
| **At which pH?** |
| **If you use enzymatic retrieval, which agent do you use?** |
| **If you use beta-catenin, what are its specifications?** |
| Clone: |
| Firm: |
| Concentration: |
| Tissue for validation: |
| **How do you perform antigen retrieval for beta-catenin?** |
| **If you perform HIER, how is it performed?** |
| **Which buffer is used?** |
| **At which pH?** |
| **If you use enzymatic retrieval, which agent do you use?** |
| **If you use p120-catenin, what are its specifications?** |
| Clone: |
| Firm: |
| Concentration: |
| Tissue for validation: |
| **How do you perform antigen retrieval for p120-catenin?** |
| **If you perform HIER, how is it performed?** |
| **Which buffer is used?** |
| **At which pH?** |
| **If you use enzymatic retrieval, which agent do you use?** |
| **Do you use another antibody other than E-cadherin, beta-catenin and/or p120-catenin?** |
| **If you use another antibody, what are its specifications?** |
| Specify antibody: |
| Clone: |
| Firm: |
| Concentration: |
| Tissue for validation: |
| **How do you perform antigen retrieval for this antibody?** |
| **If you perform HIER, how is it performed?** |
| **Which buffer is used?** |
| **At which pH?** |
| **If you use enzymatic retrieval, which agent do you use?** |
| **Do you use DNA sequencing for cases with inconclusive features?** |

**Supplementary Table 2: characteristics of participants per continent**

| | | Africa n (%) | Asia n (%) | Europe n (%) | North America n (%) | Oceania n (%) | South America n (%) | p-value |
|---|---|---|---|---|---|---|---|---|
| Center of activities | Large tertiary hospital | 3 (75.0) | 11 (40.7) | 26 (28.3) | 2 (18.2) | 1 (50.0) | 1 (9.1) | 0.1424 |
| | Private laboratory | 0 (0.0) | 1 (3.7) | 9 (9.8) | 0 (0.0) | 0 (0.0) | 4 (36.4) | |
| | Small community hospital | 0 (0.0) | 2 (7.4) | 4 (4.3) | 0 (0.0) | 0 (0.0) | 1 (9.1) | |
| | University Hospital | 1 (25.0) | 13 (48.1) | 53 (57.6) | 9 (81.8) | 1 (50.0) | 5 (45.5) | |
| Average volume breast cancer samples/year | 0-150 samples | 1 (25.0) | 4 (14.8) | 1 (1.1) | 0 (0.0) | 0 (0.0) | 2 (18.2) | 0.0005 |
| | 151-300 samples | 2 (50.0) | 10 (37.0) | 15 (16.3) | 1 (9.1) | 0 (0.0) | 1 (9.1) | |
| | 301-500 samples | 1 (25.0) | 3 (11.1) | 22 (23.9) | 0 (0.0) | 2 (100.0) | 2 (18.2) | |
| | >500 samples | 0 (0.0) | 10 (37.0) | 54 (58.7) | 10 (90.9) | 0 (0.0) | 6 (54.5) | |
| Number of pathologist handling breast cancer specimen | 1 | 0 (0.0) | 7 (25.9) | 1 (1.1) | 0 (0.0) | 0 (0.0) | 2 (18.2) | 0.0005 |
| | 1 to 4 | 0 (0.0) | 11 (40.7) | 51 (55.4) | 4 (36.4) | 0 (0.0) | 8 (72.7) | |
| | 5 or more | 4 (100.0) | 9 (33.3) | 40 (43.5) | 7 (63.6) | 2 (100.0) | 1 (9.1) | |
| Total | | 4 | 27 | 92 | 11 | 2 | 11 | |

## Supplementary Table 3: Overview of used concentration per used E-cadherin clone

| Concentration per clone | Number of participants |
|---|---|
| **NCH-38** | **38 (42%)** |
| RTU | 12 (13%) |
| 1/100 | 10 (11%) |
| 1/50 | 4 (4%) |
| 1/200 | 3 (3%) |
| 1/300 | 1 (1%) |
| 1/50 to 1/100 | 1 (1%) |
| 1/170 | 1 (1%) |
| 1/25 | 1 (1%) |
| Missing data | 5 (5%) |
| **Clone 36** | **15 (16%)** |
| RTU | 8 (9%) |
| 0,314 µg/ml | 2 (2%) |
| 1/200 | 1 (1%) |
| Missing data | 4 (4%) |
| **EP700Y** | **14 (15%)** |
| 1/200 | 4 (4%) |
| RTU | 5 (5%) |
| 0,314 µg/ml | 1 (1%) |
| 1/700 | 1 (1%) |
| unknown | 1 (1%) |
| Missing data | 1 (1%) |
| **Clone 36B5** | **7 (8%)** |
| RTU | 3 (3%) |
| 1/100 | 1 (1%) |
| 1/40 | 1 (1%) |
| Missing data | 2 (2%) |
| **unknown** | **6 (7%)** |
| Missing data | 6 (7%) |
| **4A2C7** | **5 (5%)** |
| 57µg/l | **1 (1%)** |
| 1,25 µg/ml | **1 (1%)** |
| 1/50 | **1 (1%)** |
| 570µg/l | **1 (1%)** |
| Missing data | **1 (1%)** |
| **HECD1** | **1 (1%)** |

| | |
|---|---|
| 1/50 | **1 (1%)** |
| **IHC564** | **1 (1%)** |
| Unknown | **1 (1%)** |
| **EP6** | **1 (1%)** |
| 1/1000 | **1 (1%)** |
| **ECH-6** | **1 (1%)** |
| 1/100 | **1 (1%)** |
| **G-10** | **1 (1%)** |
| 1/100 | **1 (1%)** |
| **GM016** | **1 (1%)** |
| RTU | **1 (1%)** |
| **Grand Total** | **91** |

## Supplementary Table 4: E-cadherin antibody clone per continent

| | |
|---|---|
| **Europe** | **92** |
| Missing data | 36 |
| NCH-38 | 24 |
| EP700Y | 11 |
| Clone 36 | 10 |
| Clone 36B5 | 5 |
| 4A2C7 | 2 |
| ECH-6 | 1 |
| HECD1 | 1 |
| GM016 | 1 |
| G-10 | 1 |
| **Asia** | **27** |
| NCH-38 | 8 |
| Missing data | 8 |
| unknown | 4 |
| 4A2C7 | 3 |
| Clone 36 | 2 |
| Clone 36B5 | 1 |
| IHC564 | 1 |
| **South America** | **11** |
| NCH-38 | 4 |
| Missing data | 4 |
| EP700Y | 2 |
| EP6 | 1 |
| **North America** | **11** |
| Missing data | 7 |
| Clone 36 | 2 |
| NCH-38 | 1 |
| Clone 36B5 | 1 |
| **Africa** | **4** |
| Missing data | 2 |
| EP700Y | 1 |
| NCH-38 | 1 |
| **Oceania** | **2** |
| Missing data | 1 |
| Clone 36 | 1 |
| **Grand Total** | **147** |

## Supplementary Table 5: staining procedures per antibody

| **Epitope retrieval modality** | **E-cadherin** | **β-catenin** | **p120-catenin** |
|---|---|---|---|
| Enzymatic retrieval | 2 | 1 | 0 |
| Heat induced epitope retrieval (HIER) | 76 | 31 | 23 |
| No Antigen retrieval | 10 | 4 | 5 |
| Unknown | 3 | 0 | 1 |
| **Grand Total** | **91** | **36** | **29** |
| | | | |
| **HIER modality** | **E-cadherin** | **β-catenin** | **p120-catenin** |
| Hot plate | 20 | 9 | 6 |
| Microwave | 7 | 2 | 1 |
| Pressure cooker | 4 | 6 | 2 |
| Stainer | 28 | 11 | 11 |
| Steamer | 2 | 0 | 1 |
| Water bath | 3 | 1 | 0 |
| Unknown | 11 | 1 | 2 |
| Other | 1 | 1 | 0 |
| **Grand Total** | **76** | **31** | **23** |
| | | | |
| **HIER buffer** | **E-cadherin** | **β-catenin** | **p120-catenin** |
| **Acidic** | **3** | **5** | **2** |
| Low pH (<7,0) | 3 | 5 | 2 |
| **Basic pH** | **60** | **24** | **19** |
| High pH (>8,0) | 38 | 10 | 10 |
| slightly basic pH [7,0-8,0] | 8 | 6 | 4 |
| Unknown | 14 | 8 | 5 |
| **Missing data** | **13** | **2** | **2** |
| **Grand Total** | **76** | **31** | **23** |

## Supplementary Table 6: concentration per reported clone

### Supplementary Table 6a: concentration per clone for β-catenin

| Concentration per clone | Number of participants |
|---|---|
| **Clone 14** | **20** |
| RTU | 6 |
| unknown | 5 |
| 1/100 | 2 |
| 1/200 | 2 |
| 1/75 | 1 |
| 1/150 | 1 |
| 1/300 | 1 |
| 1.25 µg/ml | 1 |
| 1/250 | 1 |
| **β-Catenin-1** | **12** |
| 1/200 | 3 |
| RTU | 2 |
| 1/1000 | 1 |
| 1/400 | 1 |
| 2.2mg/L | 1 |
| unknown | 4 |
| **17C2** | **3** |
| 1/100 | 1 |
| RTU | 1 |
| unknown | 1 |
| **CAT-5H10** | **2** |
| 1/200 | 1 |
| 1/100 | 1 |
| unknown | 2 |
| **Grand Total** | **38** |

### Supplementary Table 6b: concentration per clone for p120-catenin

| Concentration per clone | Number of participants |
|---|---|
| **Clone 98** | **14** |
| unknown | 5 |
| RTU | 3 |
| 1/500 | 1 |
| 1/200 | 1 |
| 1/150 | 1 |
| 0.076µg/mL | 1 |
| 1/250 | 1 |
| 1/300 | 1 |
| **EP66** | **7** |
| RTU | 3 |

| | |
|---|---|
| 1/1800 | 1 |
| 1/200 | 1 |
| 1/75 | 1 |
| unknown | 1 |
| **MRQ-5** | **5** |
| 1/50 | 2 |
| RTU | 1 |
| 1/100 | 1 |
| 1/400 | 1 |
| unknown | 4 |
| **15D2** | **1** |
| RTU | 1 |
| **Grand Total** | **29** |

Supplementary Table 6c: concentration per clone for other reported antibodies

| Concentration per clone | Number of participants |
|---|---|
| **Catenin Delta-1 (D7S2M)** | **1** |
| 0.7 µg/mL | 1 |
| **Cytokeratin 34βE12** | **5** |
| 1/100 | 1 |
| 1/300 | 1 |
| 1/50 | 2 |
| RTU | 1 |
| **P-cadherin (clone 56)** | **1** |
| 1/100 | 1 |
| **Grand Total** | **7** |
